# Gene therapy for Whiskott–Aldrich syndrome: The latest news

**DOI:** 10.1002/ctm2.815

**Published:** 2022-04-18

**Authors:** Marina Cavazzana, Adrian Thrasher

**Affiliations:** ^1^ Department of Biotherapy Hôpital Universitaire Necker‐Enfants Malades Groupe Hospitalier Paris Centre Assistance Publique–Hopitaux de Paris Paris France; ^2^ Biotherapy Clinical Investigation Center Groupe Hospitalier Universitaire Paris Centre Assistance Publique‐Hôpitaux de Paris Paris France; ^3^ Imagine Institute Université Paris Paris France; ^4^ Molecular and Cellular Immunology Section UCL Great Ormond Street Institute of Child Health University College London London UK

Dear Editor,

Wiskott–Aldrich syndrome (WAS) is a severe X‐linked primary immunodeficiency disorder with an incidence of between 1 in 50 000 and 1 in 250 000 live births.^1^, ^2^ The syndrome is caused by loss‐of‐function mutations in the *WAS* gene that impair or abolish expression of the WAS protein (WASp).^3^ There is a genotype‐phenotype correlation; the severity of the clinical phenotype and thus the most appropriate treatment can be predicted from the type of gene mutation and the latter's impact on WASp levels.^4^, ^5^, ^6^ The WAS severity score is based on the severity of thrombocytopenia (scored 0.5 or 1), the severity of eczema and immunodeficiency (scored from 2 to 4) and the presence of autoimmunity or malignancy (scored 5).^7^

WASp is a key regulator of the actin cytoskeleton. It coordinates the assembly of actin filaments in response to cell signalling events. The fact that WASp is only expressed in all haematopoietic cells (other than red blood cells) explains the broad range of associated clinical manifestations. Impairments in WASp function have been shown to impair multiple cellular processes in both myeloid and lymphoid lineages, including cell adhesion, cell migration, phagocytosis, immune synapse assembly,^8^ autophagy, inflammasome regulation^9^ and, more recently, the excessive activation of innate immune system cells and production of inflammatory cytokines.^10^ The pathogenesis of the platelet defect is not fully understood but is thought to involve megakaryocyte dysfunction that leads to small, abnormal platelets^11^, ^12^ and increased platelet destruction in the spleen. Profound, treatment‐refractory thrombocytopenia is the most severe, life‐threatening early‐onset form of WAS.^13^ In contrast, X‐linked thrombocytopenia is a milder disease entity that can only be diagnosed after the age of two (i.e., if the signs and symptoms remain mild after this age, with no disease progression).

It is generally accepted that children lacking WASp are candidates for curative allogeneic haematopoietic stem cell transplantation (HSCT) or (if available) gene therapy via genetically modified autologous stem cell transplantation. In fact, patients who fully lack WASp do not survive beyond their second or third decade of life.^14^ In 1968, WAS was the first primary immunodeficiency to be cured via HSCT.^15^ Since then, several large, retrospective analyses have evidenced impressive improvements in overall and event‐free survival rates.

In the two largest studies recently performed by the Primary Immune Deficiency Treatment Consortium^16^ and the European Bone Marrow Transplantation/European Society for Immunodeficiency,^17^ the overall survival rates were 91% and 88.7%, respectively. Both studies found that age at transplantation had a strong impact on the outcome. Patients under the age of 5 at the time of HSCT had a significantly better overall survival rate. The importance of achieving full chimerism was confirmed in both studies because platelet counts were higher in patients who achieved full (> 95%) donor myeloid engraftment than in those with low‐level (5%‐49%) engraftment. Full chimerism also appears to reduce the risk of post‐HSCT autoimmunity.

How does HSCT compare with gene therapy? Several trials of gene therapy for classical WAS are ongoing. Although early studies were hampered by late‐onset haematological malignancies due to insertional mutagenesis,^18^ the use of third‐generation self‐inactivating lentiviral vectors has improved safety. The overall and event‐free survival rates in treated patients compare favourably with those in HSCT. Four centres (in Milan, London, Paris, and Boston) have treated 34 patients: 31 survived and have shown significant overall reductions in symptoms.^19^ In the first cohort of 15 patients (treated in Paris and Milan), seven were aged over 5 years and had a clinical score > 4. Six of the seven showed correction of autoimmunity and a reduction in the number of infections and bleeding episodes. These outcomes are better than for the largest study of allogeneic HSCT to date, in which the overall survival rate in this age group was 66%.

Of course, this enthusiasm should be tempered by the small number of patients who have undergone gene therapy and the variable correction of the platelet count reported to date. In our small cohort, only the patients with high levels of engraftment with transduced cells achieved a normal platelet count. All the others had a count below 50 000/mm^3^. Two parameters govern the level of myeloid chimerism observed after gene therapy: the intensity of the conditioning regimen and the level of correction of the gene‐modified autologous CD34^+^ cells. It may therefore be beneficial to intensify conditioning if toxicity can be abrogated. Monoclonal antibodies against B cells and plasmacytoid cells can further deplete the autoimmune compartment without increasing toxicity, and monoclonal antibodies against stem cells (whether conjugated to various drugs or not) might also improve these results. Nevertheless, patients with the same level of gene correction in the drug product can differ in long‐term engraftment of corrected cells, possibly as a result of damage to stem cells through chronic inflammation and autoimmunity. This question should be investigated further to improve the overall outcome and platelet correction. Last, impressive results in the absence of adverse events have been obtained in two adults treated by gene therapy. Moreover, thymic function was maintained in these two patients, showing that the immune compartment can be successfully rebuilt. Gene therapy might therefore be a particularly attractive alternative to allogeneic transplantation in older patients with severe disease.
